# Supplementary material for: Obesity increases genomic instability at DNA repeat-mediated endogenous mutation hotspots
Source: Nat Commun. 2024 Jul 23;15:6213. doi: 10.1038/s41467-024-50006-8 (PMC11266421; doi:10.1038/s41467-024-50006-8)
Supplement: Supplementary file 1 — Supplementary Information [file 41467_2024_50006_MOESM1_ESM.pdf]

Supplementary Information

Obesity increases genomic instability at DNA repeat-mediated endogenous mutation hotspots.

Pallavi Kompella[1], Guliang Wang[1], Russell E. Durrett[2], Yanhao Lai[3], Celeste Marin[3], Yuan Liu[3], Samy L. Habib[4], John DiGiovanni[1], and Karen M. Vasquez[1]*

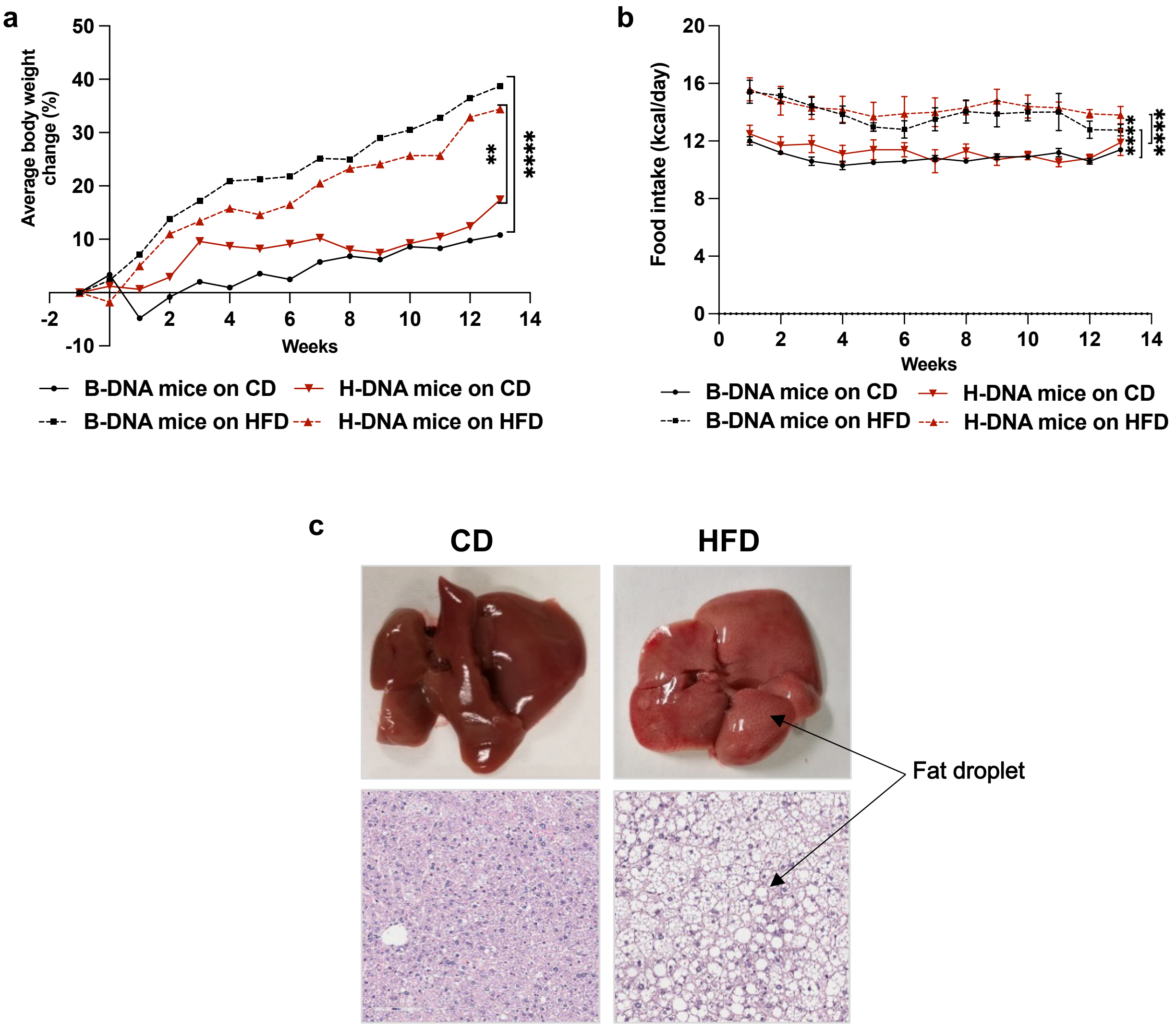

Supplementary Figure 1. High-fat diet induces obesity and liver steatosis. a Average body weight change (%) of B-DNA mice (CD, N=5; HFD, N=5), and H-DNA mice (CD, N=4; HFD, N=5) over 13 weeks. $F_{(3,56)}$ =13.25, p<0.0001. B-DNA mice CD vs. HFD: ****, p<0.0001; H-DNA mice CD vs. HFD: **, p=0.0062. **b** Average food intake (kcal/day) of B-DNA mice (CD, N=5; HFD, N=5), and H-DNA mice (CD, N=4; HFD, N=5) over 13 weeks. $F_{(3,48)}$ =100.5, p<0.0001. B-DNA mice CD vs. HFD: ****, p<0.0001; H-DNA mice CD vs. HFD: **, p=0.0062. Error bars represent mean ± SEM. Statistical analysis was performed using one-way ANOVA followed by Sidak multiple comparison test with a single pooled variance. The p value is adjusted to account for multiple comparisons with confidence interval at 95%. **c** Representative microphotographs of isolated liver tissue (top panel) and H & E-stained liver sections (bottom panel) from B-DNA mice on the CD and the HFD.

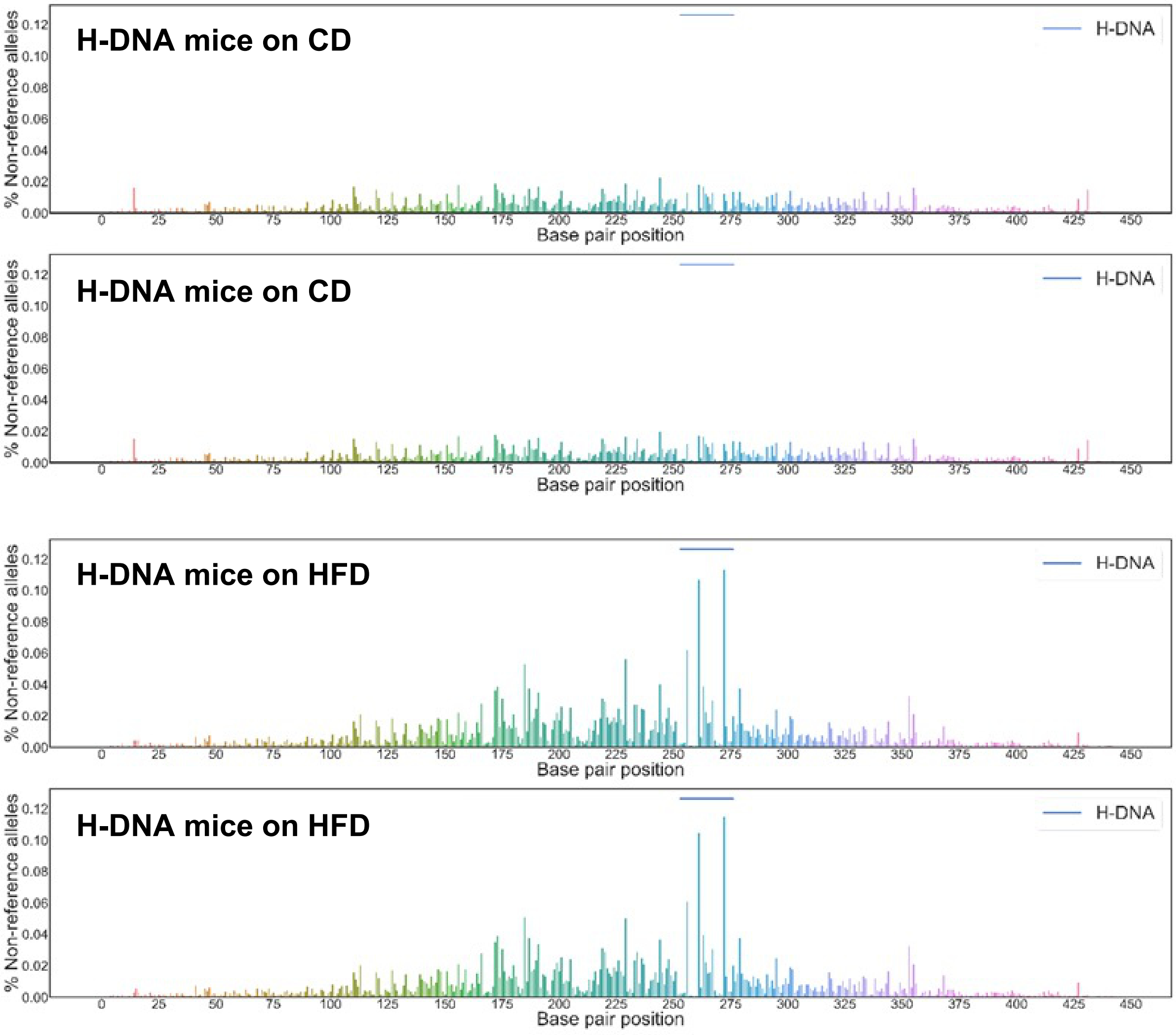

Supplementary Figure 2. Mutations mapped to the H-DNA-forming region. Illumina Mi-Seq NGS deep sequencing across a 446-bp mutation-reporter amplicon derived from genomic DNA from liver tissue of two additional H-DNA mice on the CD and the HFD shows percent non-reference alleles. The blue bar represents the H-DNA-forming region (253-276 bp) within the amplicon. The 446-bp amplicon corresponds to the 686-1132 bp region of the p2RT mutation reporter containing the H-DNA-forming sequence.

Supplementary Note 1. Statistical data analysis for Figure 2. Statistical analysis was performed using two-way ANOVA for significance in interaction (diet x B-DNA/H-DNA) followed by Sidak multiple comparison test for diet factor (CD vs. HFD) and B-DNA vs. H-DNA factor. The p value is adjusted to account for multiple comparisons with family-wise alpha threshold and confidence interval of 95%. p>0.05 (not significant), p<0.05 (*), p<0.01 (**), p<0.001 (***), p<0.0001 (****).

a Mutation frequency (x10-4) in liver tissue:
interaction: $F_{(1,15)}$=62.28, p<0.0001.
diet factor: $F_{(1,15)}$=188.5, p<0.0001 [B-DNA: p=0.0014, H-DNA: p<0.0001].
B-DNA vs. H-DNA factor: $F_{(1,15)}$=758.5, p<0.0001 [CD: p<0.0001, HFD: p<0.0001].

b Frequencies of point mutations ($x10^{-4}$) in liver tissue:
interaction: $F_{(1,15)}$=7.858, p=0.0134.
diet factor: $F_{(1,15)}$=6.886, p=0.0192 [B-DNA: p=0.9896, H-DNA: p=0.0040].
B-DNA vs. H-DNA factor: $F_{(1,15)}$=16.98, p=0.0009 [CD: p=0.6150, HFD: p=0.0003].

c Frequencies of large deletions ($x10^{-4}$) in liver tissue:
interaction: $F_{(1,15)}$=6.352, p=0.0235.
diet factor: $F_{(1,15)}$=23.05, p<0.0002 [B-DNA: p=0.2207, H-DNA: p=0.0003].
B-DNA vs. H-DNA factor: $F_{(1,15)}$=46.61, p<0.0001 [CD: p=0.0194, HFD: p<0.0001].

d Mutation frequencies ($x10^{-4}$) in brain tissue:
interaction: $F_{(1,15)}$=339.0, p<0.0001.
diet factor: $F_{(1,15)}$=479, p<0.0001 [B-DNA: p=0.0454, H-DNA: p<0.0001].
B-DNA vs. H-DNA factor: $F_{(1,15)}$=485.2, p<0.0001 [CD: p=0.0499, HFD: p<0.0001].

e Frequencies of point mutations ($x10^{-4}$) in brain tissue:
interaction: $F_{(1,15)}$=3.074, p=0.0999.
diet factor: $F_{(1,15)}$=27.34, p=0.0001 [B-DNA: p=0.0454, H-DNA: p=0.0005].
B-DNA vs. H-DNA factor: $F_{(1,15)}$=1.261, p=0.2791 [CD: p=0.8918, HFD: p=0.1040].

f Frequencies of large deletions ($x10^{-4}$) in brain tissue:
interaction: $F_{(1,15)}$=1147, p<0.0001.
diet factor: $F_{(1,15)}$=1382, p<0.0001 [B-DNA: p=0.0575, H-DNA: p<0.0001].
B-DNA vs. H-DNA factor: $F_{(1,15)}$=1727, p<0.0001 [CD: p=0.0002, HFD: p<0.0001].

g Mutation frequencies ($x10^{-4}$) in testes tissue:
interaction: $F_{(1,15)}$=404, p<0.0001.
diet factor: $F_{(1,15)}$=802.3, p<0.0001 [B-DNA: p<0.0001, H-DNA: p<0.0001].
B-DNA vs. H-DNA factor: $F_{(1,15)}$=685.3, p<0.0001 [CD: p=0.0016, HFD: p<0.0001].

h Frequencies of point mutations ($x10^{-4}$) in testes tissue:
interaction: $F_{(1,15)}$=3.429, p=0.0838.
diet factor: $F_{(1,15)}$=38.60, p<0.0001 [B-DNA: p<0.0001, H-DNA: p=0.0180].
B-DNA vs. H-DNA factor: $F_{(1,15)}$=0.0022, p=0.9629 [CD: p=0.3783, HFD: p=0.3730].

i Frequencies of large deletions ($x10^{-4}$) in testes tissue:
interaction: $F_{(1,15)}$=519.6, p<0.0001.
diet factor: $F_{(1,15)}$=632.8, p<0.0001 [B-DNA: p=0.2005, H-DNA: p<0.0001].
B-DNA vs. H-DNA factor: $F_{(1,15)}$=836.9, p<0.0001 [CD: p=0.0015, HFD: p<0.0001].

Supplementary Note 2. Statistical data analysis for Figure 7 b, c Quantitation of blots represented as violin plots with all data points and dotted lines indicating median and quartile. Statistical analysis was performed using unpaired, two-tailed Mann–Whitney U test with 95% confidence interval. p>0.05 (ns), p<0.05 (*), p<0.01 (**). For each protein, multiple comparisons were performed for significance in diet factor (CD vs. HFD) and B-DNA vs. H-DNA factor.

Ku 70 - diet factor: B-DNA (p=0.0079), H-DNA (p=0.0159).
B-DNA vs. H-DNA factor: CD (p=0.0159), HFD (p=0.0079).

DNA-PK - diet factor: B-DNA (p=0.2222), H-DNA (p=0.0159).
B-DNA vs. H-DNA factor: CD (p=0.1905), HFD (p=0.0079).

XRCC4 - diet factor: B-DNA (p=0.0952), H-DNA (p=0.7302).
B-DNA vs. H-DNA factor: CD (p=0.0159), HFD (p=0.6905).

Ligase IV - diet factor: B-DNA (p=0.0556), H-DNA (p=0.2857).
B-DNA vs. H-DNA factor: CD (p=0.0635), HFD (p=0.2222).

MRE 11 - diet factor: B-DNA (p=0.0079), H-DNA (p=0.0159).
B-DNA vs. H-DNA factor: CD (p=0.0159), HFD (p=0.2222).

RAD50 - diet factor: B-DNA (p=0.0159), H-DNA (p=0.9048).
B-DNA vs. H-DNA factor: CD (p=0.0159), HFD (p=0.0079).

XRCC1 - diet factor: B-DNA (p=0.1508), H-DNA (p>0.9999).
B-DNA vs. H-DNA factor: CD (p>0.9999), HFD (p=0.0159).

Ligase III - diet factor: B-DNA (p=0.0079), H-DNA (p=0.0317).
B-DNA vs. H-DNA factor: CD (p=0.0159), HFD (p=0.0556).
