# Supplementary material for: Obesity increases genomic instability at DNA repeat-mediated endogenous mutation hotspots
Source: Nat Commun. 2024 Jul 23;15:6213. doi: 10.1038/s41467-024-50006-8 (PMC11266421; doi:10.1038/s41467-024-50006-8)
Supplement: Supplementary file 3 — Description of Additional Supplementary Files [file 41467_2024_50006_MOESM3_ESM.pdf]

## Description of Additional Supplementary Files

File Name: Supplementary Data 1
Description: **Mutation spectra of mutation-reporter DNA from liver tissue.** Different types of point mutations (transversions, transitions, and single-base deletions) and deletions (small and large) are presented with their respective base pair position on the mutation reporters rescued from B-DNA mice on the CD (N=5, sequences analyzed: 46) and the HFD (N=5, sequences analyzed: 48); H-DNA mice on the CD (N=4, sequences analyzed: 49) and the HFD (N=5, sequences analyzed: 50).

File Name: Supplementary Data 2
Description: **Mutation spectra of mutation-reporter DNA from brain tissue.** Different types of point mutations (transversions, transitions, and single-base deletions) and deletions (small and large) are presented with their respective base pair position on the reporters rescued from B-DNA mice on the CD (N=5, sequences analyzed: 50) and the HFD (N=5, sequences analyzed: 50); H-DNA mice on the CD (N=4, sequences analyzed: 50) and the HFD (N=5, sequences analyzed: 50).

File Name: Supplementary Data 3
Description: **Mutation spectra of mutation-reporter DNA from testes tissue.** Different types of point mutations (transversions, transitions, and single-base deletions) and deletions (small and large) are presented with their respective base pair position on the reporters rescued from B-DNA mice on the CD (N=5, sequences analyzed: 46) and the HFD (N=5, sequences analyzed: 47); H-DNA mice on the CD (N=4, sequences analyzed: 47) and the HFD (N=5, sequences analyzed: 49).

File Name: Supplementary Data 4
Description: **Oligonucleotides used in the study.** CE, compatible end; DSB, DNA double-strand break; LM-PCR, Linker-mediated PCR; NCE, non-compatible end; NGS, Next generation sequencing; PCR, Polymerase chain reaction.
