# Supplementary material for: Obesity increases genomic instability at DNA repeat-mediated endogenous mutation hotspots
Source: Nat Commun. 2024 Jul 23;15:6213. doi: 10.1038/s41467-024-50006-8 (PMC11266421; doi:10.1038/s41467-024-50006-8)
Supplement: Supplementary file 8 — Reporting Summary [file 41467_2024_50006_MOESM8_ESM.pdf]

nature portfolio

Corresponding author(s): Karen M Vasquez

Last updated by author(s): Aug 31, 2023

Reporting Summary

Nature Portfolio wishes to improve the reproducibility of the work that we publish. This form provides structure for consistency and transparency in reporting. For further information on Nature Portfolio policies, see our Editorial Policies and the Editorial Policy Checklist.

Statistics

For all statistical analyses, confirm that the following items are present in the figure legend, table legend, main text, or Methods section.

n/a	Confirmed	
☐	☒	The exact sample size (*n*) for each experimental group/condition, given as a discrete number and unit of measurement
☐	☒	A statement on whether measurements were taken from distinct samples or whether the same sample was measured repeatedly
☐	☒	The statistical test(s) used AND whether they are one- or two-sided<br>*Only common tests should be described solely by name; describe more complex techniques in the Methods section.*
☒	☐	A description of all covariates tested
☐	☒	A description of any assumptions or corrections, such as tests of normality and adjustment for multiple comparisons
☐	☒	A full description of the statistical parameters including central tendency (e.g. means) or other basic estimates (e.g. regression coefficient) AND variation (e.g. standard deviation) or associated estimates of uncertainty (e.g. confidence intervals)
☐	☒	For null hypothesis testing, the test statistic (e.g. *F*, *t*, *r*) with confidence intervals, effect sizes, degrees of freedom and *P* value noted<br>*Give P values as exact values whenever suitable.*
☒	☐	For Bayesian analysis, information on the choice of priors and Markov chain Monte Carlo settings
☒	☐	For hierarchical and complex designs, identification of the appropriate level for tests and full reporting of outcomes
☒	☐	Estimates of effect sizes (e.g. Cohen's *d*, Pearson's *r*), indicating how they were calculated

Our web collection on statistics for biologists contains articles on many of the points above.

Software and code

Policy information about availability of computer code

Data collection	a) The mutant sequences were analyzed by comparing them with the respective reference sequences using the NCBI Basic Local Alignment Search Tool (BLAST).<br>b) The Next Generation Sequencing (NGS) data was collected using the Illumina MiSeq platform at the DNA sequencing facility of the University of Texas MD Anderson Cancer Center, Smithville, Texas.<br>c) Tissue sections with immunostaining for 8-oxo-dG were imaged using NIKON-TiuNIS-Elements microscope<br>d) Gel images for DSB end-joining repair assay were captured using ChemiDoc MP (Bio-Rad, Hercules, CA) biomolecular imager
Data analysis	a) The Next Generation Sequencing (NGS) data was analyzed as follows:<br>1) After demultiplexing the samples, the millions of overlapping reads obtained from the 300 bp paired-end Illumina sequencing were aligned and merged using the Pandaseq program (Masella AP et al. 2012) with the ea_util merging algorithm.<br>2) The resulting high-quality consensus reads (400-500) were aligned to the reference sequence with bowtie2, converted to bam, and sorted.<br>3) The bamtools piledriver (Quilan A, 2014) was utilized to calculate per-base reference and non-reference alleles.<br>4) This alternate allele frequency was visualized across the amplicon reference sequence using a Python-based Jupyter Notebook and matplotlib library.<br>b) Statistical data analysis for figures 2, 4, 5, 6 and 7 was performed using Graphpad Prism software v9.0<br>c) Densitometry analysis was performed using NIH ImageJ 1.8.0_172 software with the band/peak quantification macro tool

For manuscripts utilizing custom algorithms or software that are central to the research but not yet described in published literature, software must be made available to editors and reviewers. We strongly encourage code deposition in a community repository (e.g. GitHub). See the Nature Portfolio guidelines for submitting code & software for further information.

Data

Policy information about availability of data

All manuscripts must include a data availability statement. This statement should provide the following information, where applicable:

- Accession codes, unique identifiers, or web links for publicly available datasets
- A description of any restrictions on data availability
- For clinical datasets or third party data, please ensure that the statement adheres to our policy

All data supporting the findings of this study are available within the paper and its Supplementary Information. The experimental datasets and other relevant information are available in Figshare with the identifier https://doi.org/10.6084/m9.figshare.23820834

Research involving human participants, their data, or biological material

Policy information about studies with human participants or human data. See also policy information about sex, gender (identity/presentation), and sexual orientation and race, ethnicity and racism.

Reporting on sex and gender	*Use the terms sex (biological attribute) and gender (shaped by social and cultural circumstances) carefully in order to avoid confusing both terms. Indicate if findings apply to only one sex or gender; describe whether sex and gender were considered in study design; whether sex and/or gender was determined based on self-reporting or assigned and methods used. Provide in the source data disaggregated sex and gender data, where this information has been collected, and if consent has been obtained for sharing of individual-level data; provide overall numbers in this Reporting Summary. Please state if this information has not been collected. Report sex- and gender-based analyses where performed, justify reasons for lack of sex- and gender-based analysis.*
Reporting on race, ethnicity, or other socially relevant groupings	*Please specify the socially constructed or socially relevant categorization variable(s) used in your manuscript and explain why they were used. Please note that such variables should not be used as proxies for other socially constructed/relevant variables (for example, race or ethnicity should not be used as a proxy for socioeconomic status). Provide clear definitions of the relevant terms used, how they were provided (by the participants/respondents, the researchers, or third parties), and the method(s) used to classify people into the different categories (e.g. self-report, census or administrative data, social media data, etc.) Please provide details about how you controlled for confounding variables in your analyses.*
Population characteristics	*Describe the covariate-relevant population characteristics of the human research participants (e.g. age, genotypic information, past and current diagnosis and treatment categories). If you filled out the behavioural & social sciences study design questions and have nothing to add here, write "See above."*
Recruitment	*Describe how participants were recruited. Outline any potential self-selection bias or other biases that may be present and how these are likely to impact results.*
Ethics oversight	*Identify the organization(s) that approved the study protocol.*

Note that full information on the approval of the study protocol must also be provided in the manuscript.

Field-specific reporting

Please select the one below that is the best fit for your research. If you are not sure, read the appropriate sections before making your selection.

☒ Life sciences ☐ Behavioural & social sciences ☐ Ecological, evolutionary & environmental sciences

For a reference copy of the document with all sections, see nature.com/documents/nr-reporting-summary-flat.pdf

Life sciences study design

All studies must disclose on these points even when the disclosure is negative.

Sample size	Sample size was determined based on similar studies in this field.
Data exclusions	In Fig. 5d (B-DNA mice on HFD) mouse #5 has been removed from the figure for clarity and excluded from data analysis. The reason is that the majority of the input substrate remained intact due to our inadvertent omission of the DNA substrate incubation in the tissue extract.
Replication	To ensure robust reproducibility, 4-5 biological replicates were included for data collection and analysis for all the experiments in the study. Due to the high cost, 3 biological replicates were included for Next Generation sequencing data collection and analysis
Randomization	The transgenic B-DNA and H-DNA mice used in the study were randomized into control diet or high-fat diet group based on body weight before the initiation of the study.
Blinding	The investigators performing data acquisition and analysis for Next Generation sequencing were blinded to group allocation. Blinding could not be performed for other experiments in the study as it required data collection and analysis from two types of transgenic mice on two

different diets and three different tissues which could lead to the risk of mislabeling.

Reporting for specific materials, systems and methods

We require information from authors about some types of materials, experimental systems and methods used in many studies. Here, indicate whether each material, system or method listed is relevant to your study. If you are not sure if a list item applies to your research, read the appropriate section before selecting a response.

Materials & experimental systems

n/a	Involved in the study
☐	☒ Antibodies
☒	☐ Eukaryotic cell lines
☒	☐ Palaeontology and archaeology
☐	☒ Animals and other organisms
☒	☐ Clinical data
☒	☐ Dual use research of concern
☒	☐ Plants

Methods

n/a	Involved in the study
☒	☐ ChIP-seq
☒	☐ Flow cytometry
☒	☐ MRI-based neuroimaging

Antibodies

Antibodies used

1) Mouse monoclonal anti-8-oxo-dG, Abcam, Cat# ab64548/206461, RRID: AB_1141628, Clone: 2Q2311, 1:100 dilution
2) Rabbit polyclonal anti-Mouse IgG+IgM+IgA H&L (FITC) secondary antibody, Abcam, Cat # ab8517, RRID:AB_955139, 1:200 dilution
3) Rabbit polyclonal anti-Ku70, Abclonal, Cat# A7330, RRID: AB_2767867, 1:4000 dilution
4) Mouse monoclonal anti-DNA-PK, Invitrogen, Cat #MA5-13238, RRID: AB_2227681, Clone: 18-2, 1:500 dilution
5) Rabbit polyclonal anti-XRCC4, Genetex, Cat# GTX109632, RRID: AB_1952609, 1:500 dilution
6) Rabbit polyclonal anti-DNA Ligase IV, Novus Biologicals, Cat# NBP2-16182, 1:500 dilution
7) Rabbit monoclonal anti-XRCC1, Abclonal, Cat# A4135, RRID: AB_2863192, Clone: ARC0915, 1:500 dilution
8) Mouse monoclonal anti-DNA Ligase III, Novus Biologicals, Cat# NBP1-41190, Clone: 1F3, 1:500 dilution
9) Rabbit polyclonal anti-MRE11, Novus Biologicals, Cat# NB100-142, RRID: AB_10077796, 1:1000 dilution
10) Rabbit polyclonal anti-RAD50, Genetex, Cat# GTX32832, RRID:AB_10077796, 1:500 dilution
11) Rabbit polyclonal anti-gammaH2AX (Ser139), Bethyl Laboratories, Cat# IHC00059, RRID: AB_533402, 1:5000 dilution
12) Rabbit polyclonal anti-OGG1, Novus Biologicals, Cat# NB100-106, RRID: AB_10104097, 1:500 dilution
13) Rabbit monoclonal anti-Vinculin, Cell Signaling, Cat# 13901, RRID: AB_2728768, 1:2000 dilution
14) Rabbit polyclonal anti-beta actin, enQuire Bioreagents, Cat# AB822750UG, 1:2000 dilution

Validation

1) https://www.abcam.com/products/primary-antibodies/oxoguanine-8-antibody-2q2311-ab206461.html
2) https://www.abcam.com/products/secondary-antibodies/rabbit-mouse-iggigmiga-hl-fitc-ab8517.html
3) https://abclonal.com/catalog-antibodies/Ku70RabbitpAb/A7330
4) https://www.fishersci.com/shop/products/dna-pk-monoclonal-antibody-18-2-invitrogen/PIMA513238
5) https://www.genetex.com/Product/Detail/XRCC4-antibody/GTX109632
6) https://www.novusbio.com/products/dna-ligase-iv-antibody_nbp2-16182
7) https://abclonal.com/catalog-antibodies/XRCC1RabbitmAb/A4135
8) https://www.novusbio.com/products/dna-ligase-iii-antibody-1f3_nbp1-41190
9) https://www.novusbio.com/products/mre11-antibody_nb100-142
10) https://www.genetex.com/Product/Detail/Rad50-antibody/GTX32832
11) https://www.fishersci.com/shop/products/phospho-gamma-h2ax-ser139-ihc-polyclonal-bethyl-laboratories/p-7144001
12) https://www.novusbio.com/products/ogg1-antibody_nb100-106
13) https://www.cellsignal.com/products/primary-antibodies/vinculin-e1e9v-xp-rabbit-mab/13901?_requestid=854318
14) https://www.fishersci.com/shop/products/beta-actin-ab-load-ctrl-50ug-1/NC1666557

Animals and other research organisms

Policy information about studies involving animals; ARRIVE guidelines recommended for reporting animal research, and Sex and Gender in Research

Laboratory animals

Species: Mus Musculus.
Transgenic: These mice were engineered to carry a mutation-reporter construct integrated into their chromosomes, consisting of either H-DNA or control B-DNA sequences located upstream of a lacZ mutation-reporter gene.
Background: FVB/N (JAX).
Sex: Males
Age: 5-6 weeks at study initiation.

Wild animals

The study did not involve wild animals

Reporting on sex

Scientific literature shows that male mice are more susceptible to diet-induced weight gain. Therefore, based on similar studies in this field, male mice were used for this study. The gender of the mice was determined based on the anogenital distance.

Field-collected samples	The study did not involve samples collected from the field
Ethics oversight	The study was performed in accordance to the The University of Texas at Austin Institutional Animal Care and Use Committee (IACUC) protocols AUP-2016-00286 and AUP-2019-00258.

Note that full information on the approval of the study protocol must also be provided in the manuscript.
